# Differences in level of confidence in diabetes care between different groups of trainees: the TOPDOC diabetes study

**DOI:** 10.1186/1472-6920-14-191

**Published:** 2014-09-17

**Authors:** Christopher J Smith, Jyothis T George, David Warriner, David J McGrane, Kavithia S Rozario, Hermione C Price, Emma G Wilmot, Partha Kar, Irene M Stratton, Edward B Jude, Gerard A McKay

**Affiliations:** Royal Alexandria Hospital, Paisley, PA2 9PN UK; Diabetes Trials Unit, Churchill Hospital, Headington, Oxford OX3 7LJ UK; Doncaster Royal Infirmary, Doncaster, DN2 5LT UK; Southern General Hospital, Glasgow, G51 4TF UK; Childrens’ Hospital, Oxford, OX3 9DU UK; Community Diabetes Service, Long Term Condition Centre, Hampshire, SO43 7NG UK; Queens Medical Centre, Nottingham University Hospitals, Nottingham, NG5 1PB UK; Queen Alexandria Hospital, Portsmouth, PO6 3LY UK; Gloucestershire Diabetic Retinopathy Research Group, Cheltenham General Hospital, Cheltenham, GL53 7AL UK; Tameside General Hospital, Ashton-under-Lyme, Lancashire, OL6 9RW UK; Glasgow Royal Infirmary, Glasgow, G4 0SF UK

## Abstract

**Background:**

There is an increasing prevalence of diabetes. Doctors in training, irrespective of specialty, will have patients with diabetes under their care. The aim of this further evaluation of the TOPDOC Diabetes Study data was to identify if there was any variation in confidence in managing diabetes depending on the geographical location of trainees and career aspirations.

**Methods:**

An online national survey using a pre-validated questionnaire was administered to trainee doctors. A 4-point confidence rating scale was used to rate confidence in managing aspects of diabetes care and a 6-point scale used to quantify how often trainees would contribute to the management of patients with diabetes. Responses were grouped depending on which UK country trainees were based and their intended career choice.

**Results:**

Trainees in Northern Ireland reported being less confident in IGT diagnosis, use of IV insulin and peri-operative management and were less likely to adjust oral treatment, contact specialist, educate lifestyle, and optimise treatment. Trainees in Scotland were less likely to contact a specialist, but more likely to educate on lifestyle, change insulin, and offer follow-up advice. In Northern Ireland, Undergraduate (UG) and Postgraduate (PG) training in diagnosis was felt less adequate, PG training in emergencies less adequate, and reporting of need for further training higher. Trainees in Wales felt UG training to be inadequate. In Scotland more trainees felt UG training in diagnosis and optimising treatment was inadequate. Physicians were more likely to report confidence in managing patients with diabetes and to engage in different aspects of diabetes care. Aspiring physicians were less likely to feel the need for more training in diabetes care; however a clear majority still felt they needed more training in all aspects of care.

**Conclusions:**

Doctors in training have poor confidence levels dealing with diabetes related care issues. Although there is variability between different groups of trainees according to geographical location and career aspirations, this is a UK wide issue. There should be a UK wide standardised approach to improving training for junior doctors in diabetes care with local training guided by specific needs.

## Background

There are 2.9 million people diagnosed with diabetes and an estimated 850 000 undiagnosed cases in the UK. The Association of Public Health Observatories model estimates that by 2025 there may be up to 5 million people with diabetes in the UK (4.19 million people in England, 371,000 people in Scotland, 288,000 people in Wales and although this model did not include Northern Ireland estimates for diagnosed and undiagnosed diabetes in the province for 2010 is 109,000 [unpublished data]) [1].

Currently, 10–20% of all hospital inpatients in the UK have a diagnosis of diabetes [2]. As a result of the increasing prevalence, the percentage of inpatients with diabetes is expected to rise. Therefore, trainee doctors are likely to be responsible for the care of people with diabetes, irrespective of their grade and specialty or the patient’s reasons for admission. Furthermore, specialist trainees providing acute medical care of inpatients and hospital admissions have a varying degree of training in diabetes prior to entering higher medical training and this could affect their particular skill set and confidence when dealing with diabetes related issues.

The Trainees Own Perception of Diabetes Care (TOPDOC) national survey of doctors in training has shown insufficient confidence in diagnosing and managing all aspects of diabetes care [3]. Qualitative studies assessing the inpatient experiences of people with diabetes suggest dissatisfaction with the level of care received including reports of staff not specialising in diabetes appearing to lack a basic knowledge about diabetes, leading to suboptimal care [4].

The national diabetes inpatient audit in England showed 40.0% of inpatients with diabetes had at least one diabetes-related medication error and 22% experienced at least one episode of hypoglycaemia [5]. The TOPDOC study was cited in the National Audit Office “The management of adult diabetes services in the NHS” report in May 2012 as demonstrating a need for an increased level of competence [6].

Using the TOPDOC Diabetes Study responses we aimed to test whether there was any regional or training speciality differences in confidence. There was no attempt to assess respondents’ competence. This is a post hoc analysis and not the question that the original study set out to answer so the findings are for hypothesis generation only.

## Methods

The TOPDOC study was an online survey of trainee doctors using a pre-validated questionnaire using peer-reviewed assessment tools. The protocol for the study has been previously published [7].

### Study participants

All junior doctors training in the UK (foundation and specialty) at the time of the survey were eligible to take part.

### Assessment tools

Confidence of respondents was assessed using the validated Royal College of Physicians ‘Confidence Rating’ (CR) scale [8]. The scale has four points—CR1: ‘not confident’, CR2: ‘satisfactory but lacking confidence’, CR3: ‘confident in some cases’ and CR4: ‘fully confident in most cases’. To assess how often trainees would do something in typical day-to-day practice, we used a six-point scale with narrative description in combination with numeric values. There was a choice of ‘always’ (100% of the time), ‘almost always’ (80–99% of the time), ‘often’ (50–79% of the time), ‘not very often’ (20–49% of the time), ‘rarely’ (5–19% of the time) or ‘never’ (<5% of the time). Respondents were also asked whether they felt that their undergraduate and postgraduate training was adequate and whether they felt they required further training to deal with aspects of managing patients with diabetes.

### Validation

The TOPDOC Study questionnaire was pre-validated in a four-stage process: (i) initial review by external experts in the field of diabetes, (ii) administration of initial draft on a sample cohort, (iii) revision of questionnaire based on feedback received and (iv) final external review. The validity of respondents was ensured by requesting General Medical Council numbers, and crosschecking with the medical register. This also prevented submission of duplicate forms [9].

### Recruitment

Recruitment of trainee doctors was through an extensive campaign using regional facilitators, who encouraged completion of the questionnaire through their local hospital or regional training networks, and a promotional campaign through medical news sources. All post-graduate training centres in the UK were sent promotional material highlighting the study. Online vouchers were used as incentives to respondents completing the study.

### Ethical statement

The Research Ethics Committee of Glasgow Royal Infirmary reviewed the study and the Chairman advised that no formal ethical approval was required.

### Statistical analysis

We used chi-squared tests to examine differences between groups in levels of confidence, aspects of current practice and perception of training. If there were significant differences we compared the largest group (ie England, or Physicians) against the other 3 groups individually.

## Results

### Demographics

The process followed in the validation of responses and the detailed demographics have been previously reported [3]. In brief, 2149 validated complete responses to the online questionnaire were received. All regions of the UK were represented including 312 (14.5%) responses from Scotland, 306 (14.2%) from Wales and 172 (8.0%) from Northern Ireland. The median number of years of full-time post-graduate training was two. The respondents stated it was likely they would pursue a career in the following specialties: 41% in general (internal) medicine and its subspecialties, 18% in primary care, 12% in anaesthetics, 10% in surgery, 6% in paediatrics, 4% in emergency medicine, 4% in women’s health, 2% in psychiatry and 2% in diagnostics.

### National groups

#### Confidence and clinical practice

The number of each national group who were confident (either “confident in some cases” or “confident in most cases”) in relation to 8 different aspects of diabetes diagnosis and management is shown in Table 1. Trainees from Northern Ireland reported being less confident in IGT diagnosis, use of IV insulin and peri-operative management.

**Table 1:** **No of trainees (percentage of group) confident in different aspects of diabetes care**

Question/Number confident	NI (172)	Wales (306)	Scotland (312)	England (1359)	p value for difference between countries
Diabetes diagnosis	165(95.9%)	298(97.4%)	298(95.5%)	1276(97.4%)	0.26
IGT diagnosis	135(78.5%)	266(86.9%)	260(83.3%)	1128(86.1%)	0.035
Hypo treatment	171(99.4%)	303(99.0%)	308(98.7%)	1301(99.3%)	0.72
DKA treatment	163(94.8%)	280(91.5%)	299(95.8%)	1235(94.3%)	0.13
HONK treatment	122(70.9%)	233(76.1%)	242(77.6%)	1038(79.2%)	0.08
Use of IV insulin	125(72.7%)	256(83.7%)	272(87.2%)	1109(84.7%)	0.0003
Use of IV fluids	163(94.8%)	291(95.1%)	301(96.5%)	1254(95.7%)	0.78
Peri-operative management	124(72.1%)	249(81.4%)	254(81.4%)	1053(80.4%)	0.055

The number in each group who frequently (“always”, “almost always” or “often”) engage in a different aspect of diabetes care is shown in Table 2. Trainees in Northern Ireland were less likely to adjust oral treatment, contact specialist, educate lifestyle, and optimise treatment. Scotland based trainees were less likely to contact specialists, but more likely to educate on lifestyle, change insulin, and offer follow-up advice.

**Table 2:** **No of trainees (percentage of group) often practising different aspects of diabetes care**

Question/Number often	NI (172)	Wales (306)	Scotland (312)	England (1359)	p value for difference between countries
Often optimise	118(68.6%)	239(78.1%)	230(73.7%)	1011(77.2%)	0.051
Educate lifestyle	79(45.9%)	201(65.7%)	199(63.8%)	738(56.3%)	<0.0001
Adjust oral treatment	46(26.7%)	118(38.6%)	104(33.3%)	489(37.3%)	0.026
Change insulin	98(57.0%)	159(52.0%)	201(64.4%)	755(57.6%)	0.019
Contact specialist	108(62.8%)	292(95.4%)	277(88.8%)	1292(98.6%)	<0.0001
Offering follow up advice	108(62.8%)	228(74.5%)	234(75.0%)	896(68.4%)	0.006
Discharge planning	94(54.7%)	208(68.0%)	198(63.5%)	817(62.4%)	0.036

#### Training needs

The numbers who reported their under-graduate (UG) and post-graduate (PG) training to be adequate in relation to diagnosis or treatment optimisation in diabetes is shown in Table 3. It also shows the numbers who reported a need for further training. There were significant differences between the national groups when answering about previous training and perception of future training needs. In NI, UG and PG training in diagnosis was felt less adequate, PG training in emergencies less adequate, and reporting of need for further training was higher. Wales based trainees more likely to feel UG training to be inadequate. In Scotland more trainees felt UG training in diagnosis and optimising treatment was inadequate.

**Table 3:** **No of trainees (percentage of group) reporting training was adequate, or need for further training**

Question/Number felt training adequate, or need for further training	NI (172)	Wales (306)	Scotland (312)	England (1359)	
UG (undergraduate) training diagnosis	95(55.2%)	169(55.2%)	179(57.4%)	971(74.1%)	<0.0001
PG (postgraduate) training diagnosis	75(43.6%)	178(58.2%)	167(53.5%)	719(54.9%)	0.018
Further training diagnosis	144(83.7%)	224(73.2%)	230(73.7%)	971(74.1%)	0.043
UG training optimising	25(14.5%)	59(19.3%)	59(18.9%)	361(27.6%)	<0.0001
PG training optimising	48(27.9%)	114(37.3%)	124(39.7%)	458(35.0%)	0.061
Further training optimising	156(90.7%)	253(82.7%)	260(83.3%)	1108(84.6%)	0.10
UG emergencies	91(52.9%)	112(36.6%)	164(52.6%)	662(50.5%)	<0.0001
PG emergencies	102(59.3%)	202(66.0%)	204(65.4%)	895(68.3%)	0.11
Further training emergencies	137(79.7%)	210(68.6%)	211(67.6%)	915(69.8%)	0.033

### Training groups

#### Confidence and clinical practice

The number of each specialty group who were confident (either “confident in some cases” or “confident in most cases”) in relation to 8 different aspects of diabetes diagnosis and management is shown in Table 4. In all cases physicians were more likely to report confidence.

**Table 4:** **No of trainees confident (percentage of group) in different aspects of diabetes care**

Question/Number confident	GP (387)	Paeds (129)	Surgery (215)	Physicians (881)	p value for difference between specialties
Diabetes diagnosis	373(96.9%)	133(97.8%)	200(91.7%)	874(98.8%)	<0.0001
IGT diagnosis	322(83.6%)	111(81.6%)	172(78.9%)	801(90.5%)	<0.0001
Hypo treatment	380(98.7%)	135(99.3%)	215(98.6%)	881(99.5%)	0.32
DKA treatment	347(90.1%)	131(96.3%)	194(89.0%)	853(96.4%)	<0.0001
HONK treatment	259(67.3%)	74(54.4%)	143(65.6%)	774(87.5%)	<0.0001
Use of IV insulin	284(73.8%)	107(78.7%)	172(78.9%)	789(89.2%)	<0.0001
Use of IV fluids	358(93.0%)	130(95.6%)	206(94.5%)	867(98.0%)	0.0002
Peri-operative management	263(68.3%)	99(72.8%)	183(83.9%)	733(82.8%)	<0.0001

The number of each group who frequently (“always”, “almost always” or “often”) engage in a different aspect of diabetes care is shown in Table 5. There are significant differences between these groups. Trainees aspiring to be physicians are more likely to engage in different aspects of diabetes care. There is a non significant higher percentage of primary care trainees willing to advise on lifestyle, but not as willing to adjust oral treatment in type 2 diabetes.

**Table 5:** **No of trainees (percentage of group) often practising different aspects of diabetes care**

Question/Number often	GP (387)	Paeds (129)	Surgery (215)	Physicians (881)	
Often optimise	283(73.5%)	92(67.6%)	162(74.3%)	694(78.4%)	0.023
Educate lifestyle	251(65.2%)	86(63.2%)	108(49.5%)	526(59.4%)	0.0019
Adjust oral treatment	115(29.9%)	29(21.3%)	50(22.9%)	461(52.1%)	<0.0001
Change insulin	208(54.0%)	66(48.5%)	107(49.1%)	636(71.9%)	<0.0001
Contact specialist	374(97.1%)	131(96.3%)	215(98.6%)	834(94.2%)	0.0108
Offering follow up advice	289(75.1%)	86(63.2%)	154(70.6%)	673(76.0%)	0.0088
Discharge planning	248(64.4%)	99(72.8%)	132(60.6%)	584(66.0%)	0.1189

#### Training needs

The numbers who reported their training to be adequate in relation to diagnosis or treatment optimisation in diabetes and those who reported a need for further training is shown in Table 6. There were significant differences between the specialty groups when answering about previous training and perception of future training needs. Aspiring physicians were less likely to feel the need for more training in diabetes care, however a clear majority still felt they needed more training in all aspects of care.

**Table 6:** **No of trainees (percentage of group) reporting training was adequate, or need for further training**

Question/Number felt adequate	GP (387)	Paeds (129)	Surgery (215)	Physicians (881)	
UG training diagnosis	205(53.2%)	82(60.3%)	146(67.0%)	494(55.8%)	0.0068
PG training diagnosis	174(45.2%)	74(54.4%)	75(34.4%)	569(64.3%)	<0.0001
Require further training	335(87.0%)	116(85.3%)	164(75.2%)	618(69.8%)	<0.0001
UG training optimising	58(15.1%)	25(18.4%)	65(29.8%)	168(19.0%)	0.0002
PG training optimising	89(23.1%)	48(35.3%)	57(26.1%)	399(45.1%)	<0.0001
require Further training	370(96.1%)	124(91.2%)	178(81.7%)	720(81.4%)	<0.0001
UG emergencies	209(54.3%)	88(64.7%)	108(49.5%)	670(75.7%)	<0.0001
PG emergencies	177(46.0%)	60(44.1%)	125(57.3%)	429(48.5%)	0.032
Require further training	331(86.0%)	115(84.6%)	174(79.8%)	549(62.0%)	<0.0001

## Discussion

The TOPDOC diabetes study reported that trainee doctors, as a whole, lack sufficient confidence in dealing with diabetes related issues when caring for inpatients [3]. Further analysis of the data shows some significant, but minor, differences between different groups of trainees when looking at confidence, current practice, and perception of training needs.

Trainees based in Northern Ireland perceived a greater need for further training in diabetes care, but this must be viewed in the context that confidence is insufficient in all four national groups. Trainees in England reported greater confidence in their previous training. This highlights the inter-regional variation in confidence in training.

This analysis of the TOPDOC Diabetes study data highlights some minor variability in reported confidence and aspects of diabetes care between different groups of trainees, despite the likelihood that they will be involved in some aspect of the management of diabetes related problems in their future careers. Doctors aspiring to be physicians reported greater confidence and a lower perceived need for training. This is to be expected. However, across specialties better standards in diabetes related care are required. Although the TOPDOC study was designed primarily to ask trainees about their confidence in managing diabetes in the in patient setting some aspects of the results in the wider context of managing diabetes are of concern. For example GP trainees are confident in the diagnosis of diabetes but do not generally optimise oral treatment despite the move to greater service delivery in primary care. With the increasing prevalence of diabetes, GPs are being asked to take responsibility for routine care, allowing specialists to focus on more complex cases or sub-groups such as adolescents and antenatal diabetes. Therefore GP trainees should be more confident and proactive in dealing with diabetes-related care. It is not clear if and when GP trainees get the training that they require to support the appropriate management of diabetes in primary care, but it is likely that this will vary in its quantity and quality from region to region.

Since the study was originally published in 2011 there have been some changes to medical training including a revision of the Foundation Programme curriculum with greater emphasis on long term condition management. This in theory could have some impact on the confidence levels of current Foundation trainees but the curriculum change does not provide a template as to how training in managing long term conditions is to be delivered. Therefore, a specific UK wide strategy is required to address the concerns raised in this paper. Specialists need to be involved in leading diabetes services across the whole healthcare system and ensuring that there is appropriate training in place for GPs, trainee doctors, nurses and anyone involved in care of diabetes patients. The TOPDOC questionnaire has been shown to be a useful tool to assess doctors’ confidence levels in managing diabetes. The results from specific localities could be used to target training to address the reported training needs or nationally it could be re-administered to assess a UK wide approach to training.

## Conclusion

Trainee doctors have poor confidence levels dealing with diabetes related care issues and declare a need for more training. Although there is variability between different groups of trainees according to geographical location and career aspirations, this is a UK wide issue. The results might provide an opportunity for specialists in their own geographical locality to ensure specific aspects of training are covered e.g. targeted training of GP trainees in how to optimise oral treatment with the questionnaire re-administered to evaluate their intervention. However there should be more training in all aspects of diabetes care at both an undergraduate and postgraduate stage. For this to be successful there needs to be a UK wide standardised approach to improving training for trainee doctors in diabetes care to complement local training initiatives guided by specific needs.

## Abbreviations

TOPDOC Diabetes Study: The Trainees Own Perception of Delivery Of Care diabetes study.
